# Large Overlap Between the Intestinal and Reproductive Tract Microbiomes of Chickens

**DOI:** 10.3389/fmicb.2020.01508

**Published:** 2020-07-14

**Authors:** Naama Shterzer, Nir Rothschild, Yara Sbehat, Einav Stern, Albert Nazarov, Erez Mills

**Affiliations:** Department of Animal Sciences, Robert H. Smith Faculty of Agriculture, Food, and Environment, The Hebrew University of Jerusalem, Rehovot, Israel

**Keywords:** vertical transmission of bacteria, poultry, gut micobiota, oviduct microbiota, 16S rDNA (amplicon sequencing)

## Abstract

Recent work characterized the chicken reproductive tract (oviduct) microbiome composition and its similarity to the egg and chick microbiomes. However, the origin of the oviduct microbiome has not been addressed yet. Here, we characterized the microbiome composition along the oviduct (infundibulum, magnum, and shell gland) as well as in the gut (jejunum and cecum) of broiler breeders at 37 weeks of age of the Cobb industrial breed. We found that while the microbiome composition along the oviduct is similar, the three sites, jejunum, cecum, and oviduct hold distinct microbiomes. However, there was also a large overlap in the composition of the gut and oviduct microbiomes, with 55 and 53% of amplicon sequence variants (ASVs) representing 96 and 90% of the total abundance in the jejunum and cecum, respectively, shared with the magnum. Furthermore, we identified a strong correlation between the relative abundance of ASVs in the gut and their probability to be found in the oviduct. These results suggest that material from the gut travels the full length of the oviduct. This is possibly the result of chicken physiology which includes the cloaca, a cavity to which both the intestinal and reproductive tracts open into. As the cloaca is common to birds, reptiles, amphibians, most fish, and monotremes, our finding may be relevant to many vertebrates. Importantly, these results indicate that mere presence in, and ascending of the oviduct are not virulence characteristics specific to pathogens, as commonly thought, but are the result of chicken physiology and characterize all gut bacteria. Furthermore, whereas a vertical transmission route from the hen to the chick has been suggested, our work starts laying a mechanistic foundation to this route, by describing the movement of gut bacteria to the oviduct, where they may be enclosed in the developing egg. Last, as our results show that gut material travels the full length of the oviduct, fertilization in poultry occurs in the presence of at least bacterial products if not live bacteria, and therefore food additives, probiotics, and diet possibly have a much more direct effect on reproduction and egg formation than previously considered.

## Introduction

Efficient transfer to host progeny is crucial for the evolutionary success of gut commensals and pathogens. Furthermore, efficient vertical transmission of gut commensals to progeny is also imperative for the rapid development of progeny microbiota and the evolutionary success of the host, as the microbiota affects host development, its ability to utilize plant-derived carbohydrates, and protects the host from gut pathogens (Józefiak et al., 2004; Sommer and Bäckhed, 2013; Oakley et al., 2014).

In poultry, the vertical transmission of gut pathogens from hens to eggs and progeny has been known for some time. Pathogens, such as *Salmonella enterica* and *Campylobacter jejuni*, have been shown to colonize the oviduct, thus embedding themselves in the developing egg (Camarda et al., 2000; Gantois et al., 2009). While *Salmonella enterica* serovar Enteritidis may be able to infect the poultry ovary, most *Salmonella* serovars are thought to colonize the oviduct. Furthermore, *Salmonella* bacteria can survive the albumen environment of the egg (Lock and Board, 1992; Schoeni et al., 1995). Hence, a transmission route starting from the gut, passing through the oviduct, on the way to the egg and the chick has been established for pathogens (Berchieri et al., 2001; Gama et al., 2003; Davies and Breslin, 2004; Liljebjelke et al., 2005; Cox et al., 2012). However, it is thought that this route requires specific characteristics that are unique to pathogens.

Recently, it has been suggested that vertical transmission of commensal bacteria from hens to chicks *via* the egg exists. Ding et al. used 16S rDNA analysis to examine the microbiome composition of maternal hens’ feces, embryos, and chicks’ ceca, in three different Chinese poultry breeds (Ding et al., 2017). They showed a significant correlation between the microbiomes and defined a set of core genera possibly vertically transmitted from the hen’s gut to the chick. Lee et al. also used 16S rDNA analysis to examine the composition of the oviduct microbiome and compare it to that of the hen’s cloaca, descendent egg shell and egg white, and 18-day old embryo cecum of egg-laying Korean commercial breed hens (Lee et al., 2019). They also showed a correlation between the microbiomes at the different sites, thus establishing a possible connection between the maternal oviduct and chick gut microbiomes. Therefore, both the hen’s intestine and oviduct microbiomes were shown to possibly contribute to the chick gut microbiome. However, a comparison between the maternal hen’s intestinal and oviduct microbiomes has not been conducted.

In the present study, we characterized the composition of the gut and oviduct microbiomes in the same individual hens, of the industrial Cobb breed. We found a large overlap in the composition of the oviduct and intestinal microbiomes, and that the relative abundance of amplicon sequence variants (ASVs) in the gut correlates with the probability that they will be present in the oviduct. This suggests that gut material travels the full length of the oviduct, and that ascending the oviduct and inhabiting it in itself is not a pathogenic trait but rather a result of chicken physiology.

## Materials and Methods

### Sample Collection

All animal trials were conducted in accordance with the guidelines of the National Council for Animal Experimentation and were subjected to approval by the Hebrew University of Jerusalem’s Ethics committee, approval No. AG-19-15897-3.

Animals were brought from a commercial operation and acclimatized for 3 weeks before euthanization. GI tract and reproductive tract samples were removed from euthanized animals. GI tract contents were squeezed out and reproductive tract mucosa was scraped with a sterile glass slide. For infundibulum samples, the oviduct was cut about 1 cm into the magnum section, as this section is more rigid. The infundibulum was swabbed with a sterile swab by inserting the swab through the magnum section, avoiding contact with the magnum section, and into the infundibulum. The swab was then extracted with care through the magnum section. All samples were mixed with PBS, snap frozen with liquid nitrogen and kept at −20°C until DNA extraction.

### DNA Extraction

DNA was extracted by disruption with 0.1 mm glass beads in the presence of Tris-saturated phenol, following phenol-chloroform extraction, as described by Stevenson and Weimer (2007). Briefly, the aqueous fractions were mixed with equal volumes of phenol and separated by centrifugation. This step was repeated twice, following two aqueous phase extractions with a 1:1 (vol:vol) mixture of phenol and chloroform, and lastly, two aqueous phase extractions with chloroform. The DNA was subsequently precipitated using isopropanol precipitation and suspended in DDW.

### 16S rDNA Sequencing

16S rDNA library preparation and sequencing were performed according to the Earth Microbiome Project protocol^1^ using V4 primers 515F (GTGYCAGCMGCCGCGGTAA) and 806R (GGACTACNVGGGTWTCTAAT). Negative controls for each barcoded forward primer were also included. Thermocycling was performed under the following conditions: 94°C for 3 min, followed by 35 cycles of 94°C for 45 s, 50°C for 60 s and 72°C for 90 s, and a final step of 72°C for 10 min. Hundred and fifty base pairs paired-end sequencing was performed on an Illumina Miseq platform using a V2 reagent kit by the sequencing unit of the Faculty of Medicine at the Hebrew University of Jerusalem. Sequences were processed and taxonomy assigned using QIIME2 (Bolyen et al., 2019). ASVs were determined with Dada2 plugin version 2018.8.0 (Callahan et al., 2016) using the denoise-paired method. R2 reads were trimmed at position 2, otherwise default parameters were used. ASVs with under five reads were discarded. Taxonomy was assigned using a naive-Bayes classifier (Pedregosa et al., 2011) trained on the Greengenes database (McDonald et al., 2012). One, very abundant ASV, with the taxonomic annotation of “Bacteria” was compared to the NT database using BLAST (Altschul et al., 1990) and removed as it was 100% identical to *Gallus gallus* mitochondrion. All samples were normalized to 2,400 reads per sample (Supplementary Figure S1).

### Statistical Analysis

For richness, diversity, and within group distance measures, differences between sites were tested using Kruskal-Wallis test with Dunn’s Multiple Comparison *post-hoc* test.

Differences between whole microbiomes of the different sites were evaluated by ANOSIM using Bray-Curtis and Jaccard metrics.

Analysis of the connection between abundance in the gut and presence in the oviduct was conducted in the following manner: relative abundance was calculated for each ASV in the jejunum and cecum, from individuals in which it was present. The incidence of each ASV in the magnum was also calculated, using only individuals in which it was present in the respective gut environment. ASVs that were only found in the gut environment of one individual were omitted. Next, the data were ordered by relative abundance and binned in groups of three. This binning acted as a smoothing factor. Relative abundance and probability to appear in the magnum were averaged for each bin. A best fit semi-log nonlinear regression was identified, and Spearman correlation was calculated using GraphPad Prism version 5.03 (GraphPad Software, San Diego California USA, www.graphpad.com).

## Results

### Sample Collection and Sequencing

Oviduct samples (infundibulum swabs, and magnum and shell gland mucosa), as well as GI tract samples (jejunum and cecum digesta) were collected from 10 broiler breeders (Cobb, 37 weeks old) grown on a commercial operation and brought to the hen house at the Faculty of Agriculture, Food, and Environment of the Hebrew University of Jerusalem 3 weeks before euthanization and sample collection. Samples were analyzed by 16S rDNA amplicon sequencing to determine community structure.

### General Characteristics of Gut and Oviduct Environments

**Figure 1:**
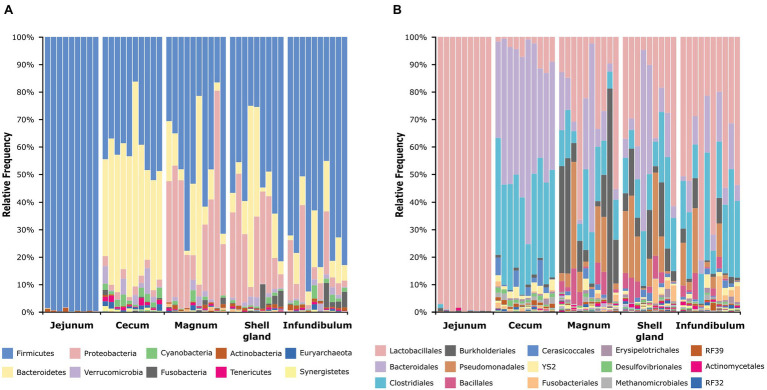
Alpha-diversity measures of the different sites sampled. **(A)** Number of observed ASVs. **(B)** Shannon diversity. Results are presented as mean ± SE; Kruskal-Wallis test with Dunn’s Multiple Comparison *post-hoc* test; ^*^0.01 ≤ *p* < 0.05, ^**^0.001 ≤ *p* < 0.01, ^***^p < 0.001.

Analysis of the richness of the sequenced samples showed the jejunum community had 17.7 ± 3.2 ASVs on average, while the cecum community had 214.8 ± 30.1 ASVs on average. In comparison, the oviduct environments had an intermediate level of richness with 142.6 ± 43.3, 118.7 ± 52.4, and 146 ± 52.7 ASVs in the shell gland, magnum, and infundibulum, respectively (Figure 1A). An analysis of diversity yielded a similar result, with the jejunum exhibiting low diversity, the cecum high diversity (2.3 ± 0.6 and 6.6 ± 0.3, respectively), and the oviduct intermediate diversity (shell gland: 5.6 ± 0.7, magnum: 5.1 ± 1.1, infundibulum: 5.4 ± 0.9; Figure 1B).

**Figure 2:**
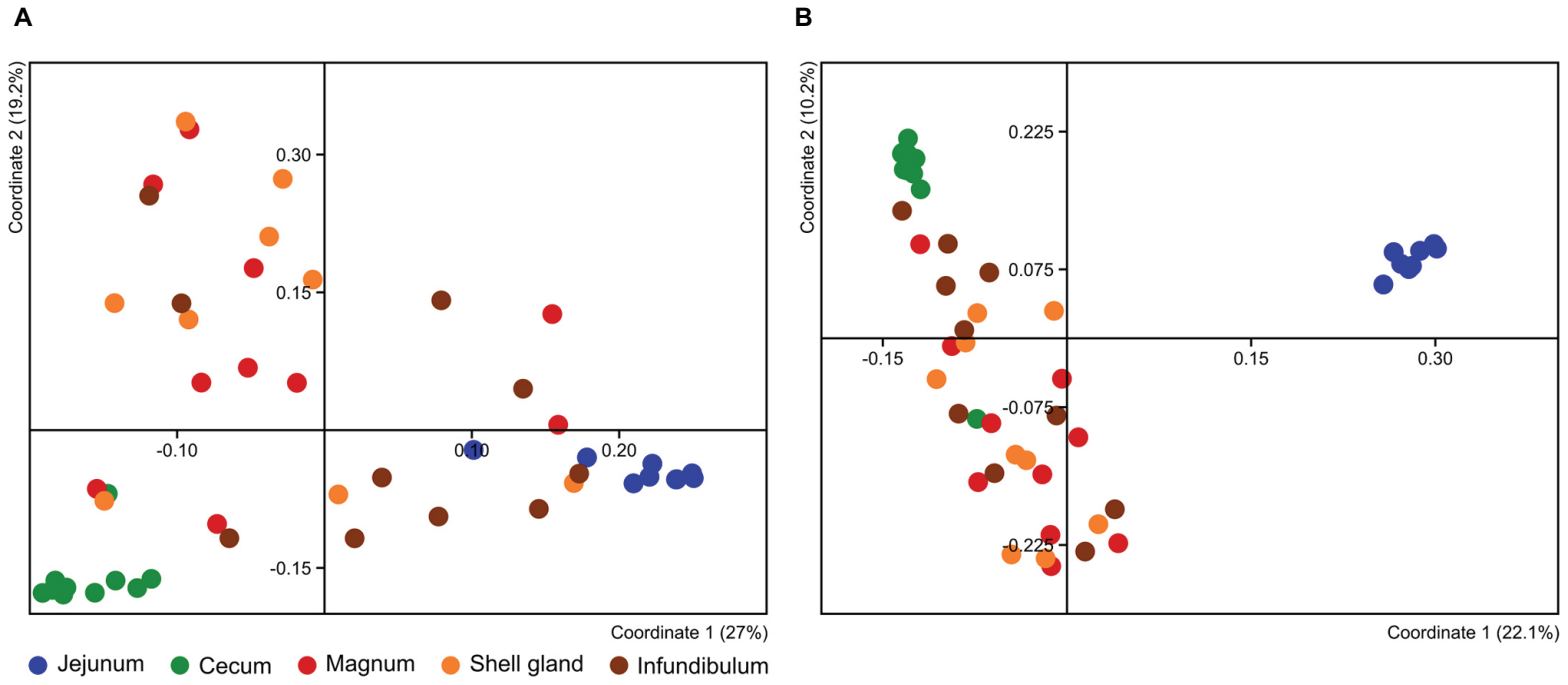
Taxonomic composition of all samples at the phylum level **(A)** and order level **(B)**. The full legends are found in Supplementary Figures S2A,B.

An analysis of the dominant phyla showed that the jejunum is dominated by *Firmicutes* (99.42 ± 0.56%), the cecum by *Firmicutes* (41.14 ± 9.98%) and *Bacteroidetes* (46.9 ± 11.9%), and the oviduct by *Firmicutes* (55.18 ± 22.34%), *Bacteroidetes* (14.47 ± 16.94%), and *Proteobacteria* (22.66 ± 18.92%; Figure 2A; Supplementary Figure S2A). On the order level, the jejunum is dominated by *Lactobacillales* (99.21 ± 0.91%), the cecum by *Bacteriodales* (46.9 ± 11.9%), *Clostridiales* (34.98 ± 7.5%), and *Lactobacillales* (5.47 ± 4.51%), while the oviduct is more diverse and dominated by *Lactobacillales* (34.78 ± 20.82%), *Bacteroidales* (14.4 ± 16.95%), *Clostridiales* (17.92 ± 9.44%), *Burkholderiales* (11.4 ± 16.53%), *Pseudomonadales* (9.81 ± 11.07%), and *Bacillales* (3.49 ± 3.64%; Figure 2B; Supplementary Figure S2B). Thus, the oviduct microbiome shares the major phyla and orders of both the jejunum and cecum while also exhibiting oviduct-unique phyla and orders.

### Microbiome Composition Along the Oviduct Is Uniform

**Figure 3:**
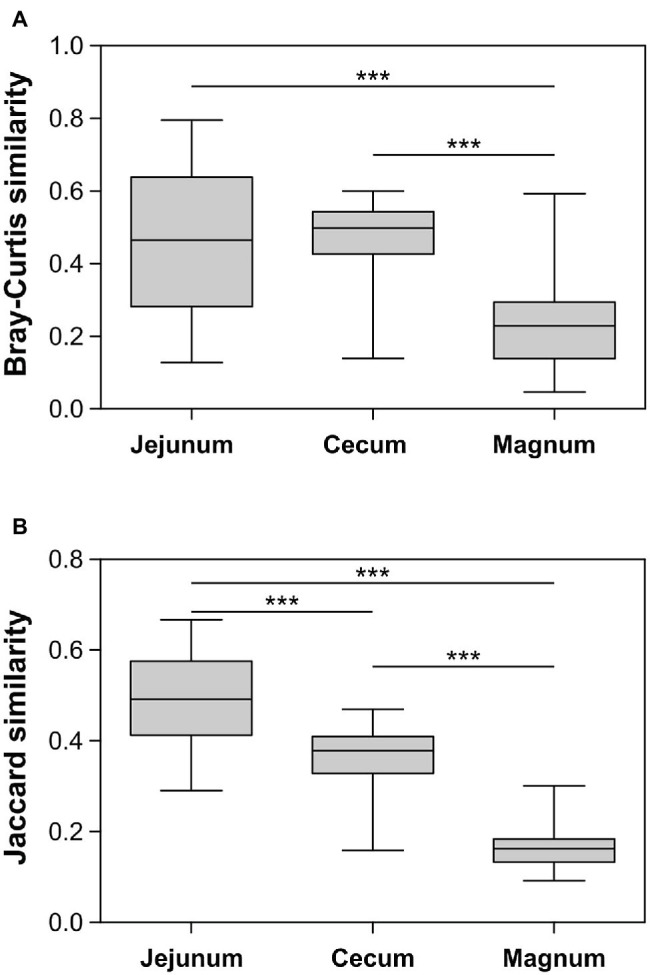
PCoA analysis of all different samples based on Bray-Curtis **(A)** and Jaccard **(B)** metrics.

To compare the different oviduct environments – infundibulum, magnum and shell gland, we performed Principal Coordinate Analysis (PCoA) based on either Jaccard distance, to compare similarity in the bacteria present, or Bray-Curtis distance, comparing also the relative abundance of the different community members. Both analyses show that the different parts of the oviduct are similar to each other (ANOSIM Bonferroni-corrected *p* > 0.1; Supplementary Tables S1, S2; Figure 3; Supplementary Figure S3). For this reason, we used the magnum environment as a representative of the whole oviduct for the rest of our analyses.

### The Oviduct and Gut Microbiomes Differ From Each Other

**Figure 4:**
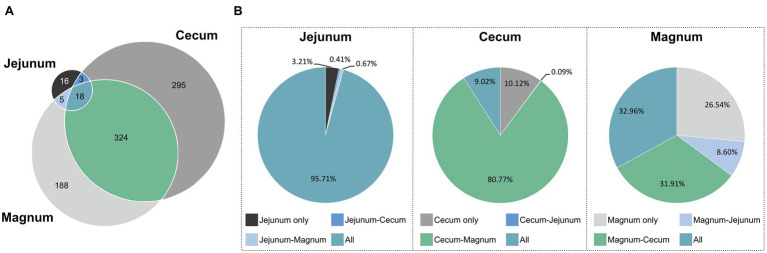
Similarity between samples within each group based on Bray-Curtis **(A)** and Jaccard **(B)** metrics. Whiskers represent minimum and maximum values; Kruskal-Wallis test with Dunn’s Multiple Comparison *post-hoc* test; ^***^p < 0.001.

The PCoA analyses also show that the cecum, jejunum, and oviduct environments are different (ANOSIM Bonferroni-corrected *p* ≤ 0.002; Supplementary Table S1; Figure 3). Furthermore, an analysis of the variance within the different environments showed that the oviduct microbiomes are more diverse than the gut environments (Figures 3, 4). Hence, the oviduct environment is different from the gut environment, not only in composition but also in variance between individuals.

### Overlap in ASVs and Abundance Between the Oviduct and Gut Environments

**Figure 5:**
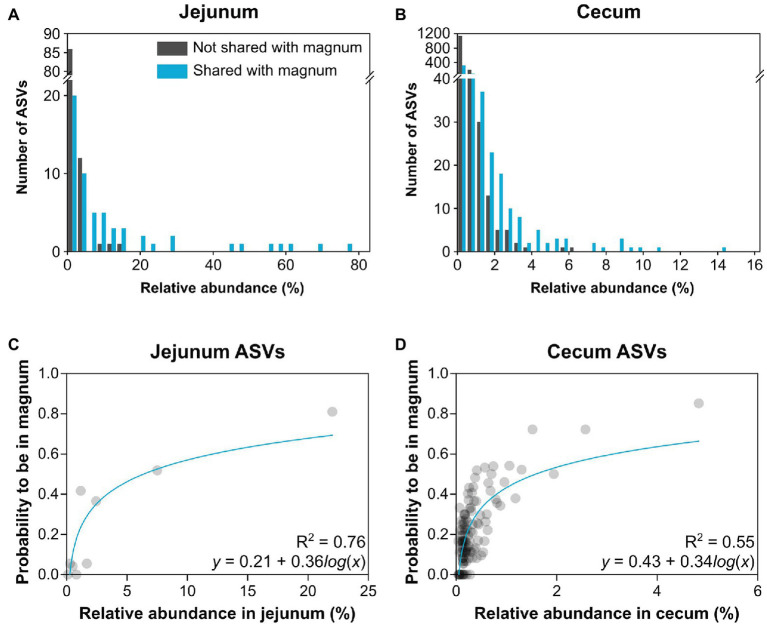
ASVs shared between the different sites. **(A)** Venn diagram of pan-microbiomes. **(B)** The total relative abundance of ASVs in each intersection, in each of the sites.

After identifying the differences between the gut and oviduct microbiomes, we wanted to characterize any similarities between these environments. First we analyzed for the pan-microbiome, including all individuals, which ASVs were shared between sites and which were unique (Figure 5A; Supplementary Figure S4). Eighteen ASVs were shared between all three sites, 324 ASVs were shared between the cecum and magnum, and another five shared between the jejunum and magnum. Thus, 53.4 and 54.8% of the cecum and jejunum ASVs, respectively, were shared with the magnum. Conversely, 64.9% of the magnum ASVs were shared with the gut sites. Thus, the gut and magnum environments exhibited a large overlap on the ASV level.

However, as different ASVs have diverse abundances in the gut and oviduct environments, ASV data may not represent all aspects of the overlap between these microbiomes. To integrate abundance into our analysis we determined the average relative abundance of each ASV in each site and summed these average abundances for each intersection of the sites (Figure 5B). We found that most of the total abundance in each site was of shared ASVs. 95.7% of the reads of the jejunum pan-microbiome, representing 18 ASVs, were of ASVs shared with both the cecum and magnum. The 16 jejunum-unique ASVs, making up 38.1% of the jejunum ASVs, only accounted for 3.2% of the jejunum reads. Similarly, the 295 cecum-unique ASVs, made up 46.1% of cecum ASVs, but represented only 10.1% of reads, while the 324 ASVs shared with the oviduct accounted for 80.8% of the cecum reads. Finally, the magnum’s 188 unique ASVs, made up 35.1% of the magnum ASVs but represented only 26.5% of the reads. The 18 ASVs shared between all three sites accounted for 33% of the magnum reads, and the 324 ASVs shared with the cecum accounted for 31.9% of the magnum reads. These results indicate that high abundance ASVs are shared, while ASVs, which are site specific and not shared, have lower abundance.

The analysis of the pan-microbiome is likely to reflect the full potential of the oviduct environment to support certain bacterial populations, but may be different from an individual level analysis, which may better represent other properties of the overlap between the gut and oviduct sites. Indeed, a comparison on the individual level yielded a lower but still clear overlap between the jejunum, cecum, and magnum of individuals (Supplementary Figure S4). On average, 52.1% of the cecum reads, representing on average 29.2% of the cecum ASVs, were shared with the same individual’s magnum. Likewise, on average, 83.6% of the jejunum reads, representing on average 36.9% of the jejunum ASVs, were shared with the individual’s magnum. Finally, on average, 47.3% of the magnum reads, representing on average 48.2% of the magnum ASVs, were shared with the individual’s jejunum and cecum. Thus, a substantial overlap between the gut and the oviduct microbiomes is present not only in the pan-microbiome but also when analyzing individual hens.

### Correlation Between the Relative Abundance of an ASV in the Gut and Probability of Its Presence in the Oviduct

**Figure 6:**
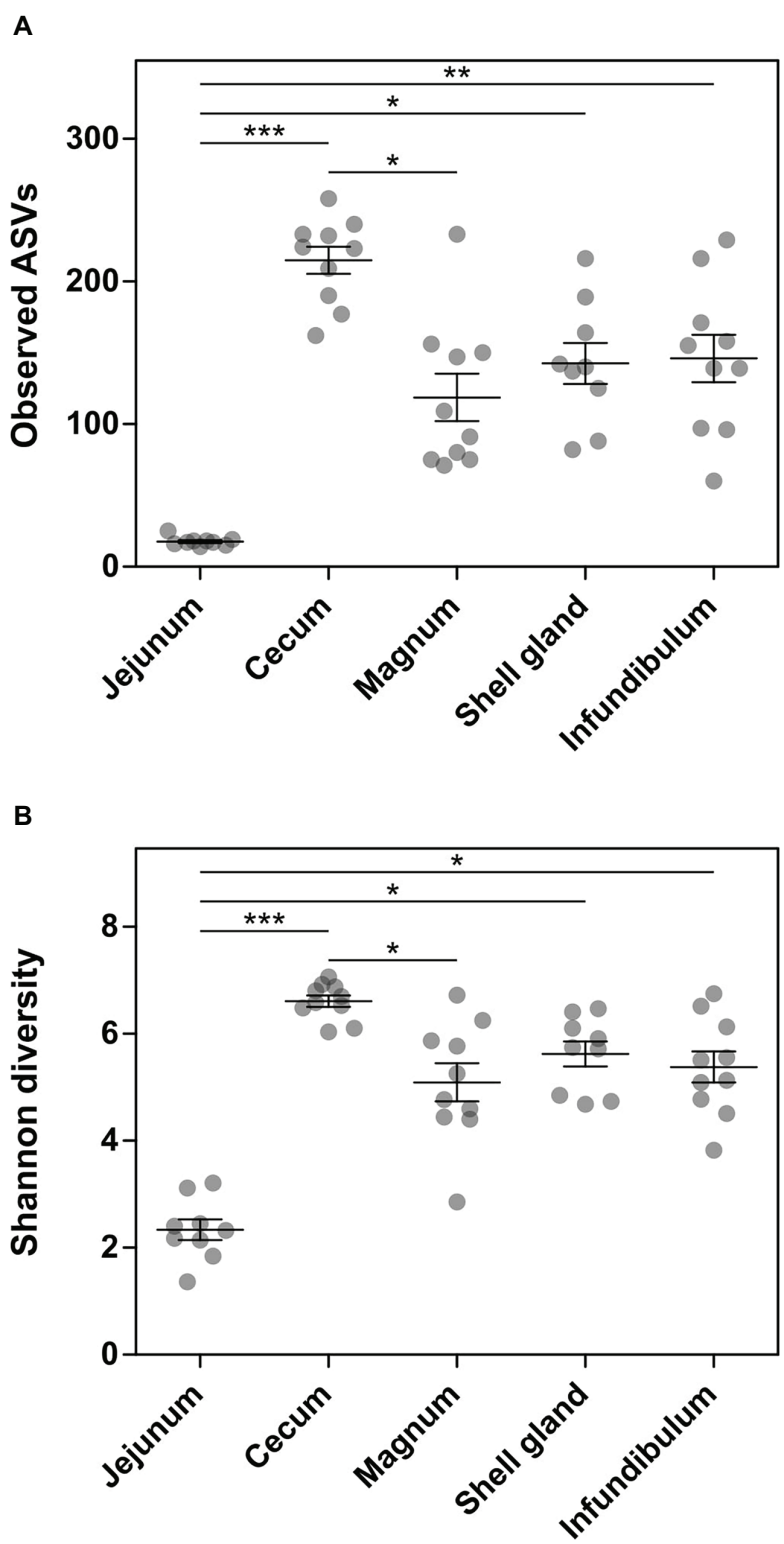
Relationship between relative abundance in the gut and presence in the oviduct. **(A,B)** Histograms describing the number of ASVs by their relative abundance of ASVs that are shared or not shared with the magnum. **(A)** Jejunum ASVs. **(B)** Cecum ASVs. **(C,D)** Nonlinear regression of the average relative abundance of bins of 3 ASVs and their average probability to be present in the magnum. **(C)** Jejunum ASVs. **(D)** Cecum ASVs.

As the overlap analysis seemed to indicate a connection between abundance of an ASV in the gut and its presence in the oviduct, we analyzed our data to better characterize this connection. First, we characterized the relative abundance of each ASV in the gut of each individual and whether it was present in the same individual’s oviduct. A comparison between the shared ASVs and ASVs that were not shared showed that as abundance in the gut increased, ASVs were more likely to be shared with the oviduct (Figures 6A,B). Furthermore, above a threshold of relative abundance in the gut, ~6.5 and ~15.25% in the cecum and jejunum, respectively, ASVs were always also present in the oviduct. To obtain a mathematical representation of these correlations we utilized the fact that we have 10 individuals to assign a “probability to be present in the oviduct” score. We found a direct correlation between the relative abundance of an ASV in the jejunum (Spearman *r* = 0.83, *p* = 0.0083, semi-log nonlinear fit *R*^2^ = 0.76; Figure 6C) and the cecum (Spearman *r* = 0.62, *p* < 0.0001, semi-log nonlinear fit *R*^2^ = 0.55; Figure 6D) and the probability that it will also be present in the oviduct. Together with the overlap between the gut and oviduct environments on both ASV and total abundance levels, these results imply that material from the gut travels to the oviduct.

## Discussion

Here, we analyzed microbiome composition in three sites along the poultry oviduct, infundibulum, magnum, and shell gland, in 10 broiler breeders of the Cobb industrial breed. Previous published work characterized the oviduct of an egg-laying Korean commercial breed (Lee et al., 2019). The oviduct microbiome of the Korean egg-laying breed was similar to the one we describe here for Cobb, and includes mostly *Firmicutes*, *Proteobacteria*, *Bacteroides*, and *Fusobacteria* (Figure 2A). At lower phylogenetic levels there is also a similarity in microbiome composition, for example, in the prominence of *Pseudomonadales* (Figure 2B). Furthermore, Lee et al. found that different sites along the oviduct were similar in microbiome composition, which we confirm here for the Cobb breed (Figure 3; Supplementary Figure S3; Supplementary Tables S1, S2). As the Cobb breed, an American poultry breed, and the egg-laying Korean breed represent different locations, breeds, and industrial focus (meat and egg laying), the fact that both have similar microbiome compositions and that sites along their oviduct have similar compositions, imply that these properties likely are true for chickens in general.

In this work, we analyzed not only the oviduct but also the cecum and jejunum microbiomes of the same individuals. This allowed us to compare the gut and oviduct microbiomes. While the oviduct and gut microbiomes were different, we could also identify similarities. Importantly, we found a large overlap between the gut microbiomes and the oviduct microbiome. This overlap was very prominent when comparing the pan-microbiome of the hens both in the ASV level as well as the total abundance level of shared ASVs (Figure 5). An analysis of this overlap in individual hens was still clear but less prominent (Supplementary Figure S4). The pan-microbiome analysis likely represents the potential of these different environments to include mostly the same bacteria, while the individual based analysis allows a snapshot of the current transient condition. Differences between the two analyses may represent differences in the retention times of matter in the different environments, additional sources of incoming bacterial strains, and/or differences in the flux of these additional sources.

In addition to the overlap in ASVs and abundances, we also found a correlation between the relative abundance of an ASV in the gut and the probability that it will also be present in the oviduct (Figure 6). Together, these results strongly imply the transfer of material from the gut to the oviduct. Furthermore, considering the fact that the microbiome is similar throughout the oviduct, material from the gut likely transfers to the oviduct and travels the full length of the oviduct up to the infundibulum.

It is possible that the overlap between the gut and oviduct microbiomes is the result of chicken physiology, and specifically the cloaca. The cloaca is a body cavity which the intestinal, urinary, and genital canals, including the oviduct, empty into. Furthermore, as sperm need to cross the cloaca and ascend the oviduct, it is possible that gut material exiting the intestinal tract into the cloaca, gets sampled into the oviduct and ascends the full length of the oviduct. As the cloaca is common to birds, reptiles, amphibians, most fish, and monotremes, it is possible that gut contents travel into the oviduct in many vertebrates.

Interestingly, it is possible that the two formulas describing the nonlinear regression which best fits the interaction between relative abundance in the gut and presence in the oviduct, are mathematical representations of poultry physiology. While the connection between the physiology and the different formula parts are currently unknown, future comparative measurements in other birds may help elucidate this connection.

While there is an overlap between the gut and oviduct environments, the oviduct environment is clearly different from the gut environment. Two properties stood out as differentiating between the oviduct and the gut environments: (a) Three orders of bacteria, *Burkholderiales*, *Pseudomonadales*, and *Bacillales*, were greatly expanded in the oviduct (Figure 2) and (b) the variance between individuals in the composition of the oviduct microbiomes compared to the gut microbiomes was larger (Figure 4). While it is likely that the input of material from the gut is a major force shaping the composition of the oviduct microbiomes, these two properties imply that there are other forces that affect the oviduct microbiome composition. These may include the oviduct environment itself, which may selectively affect the ability of different bacterial strains to survive and divide. For example, the oviduct environment includes lysozyme and other antimicrobials and is likely an aerobic environment, unlike the cecum, which is anaerobic. Indeed, the three orders of bacteria expanded in the oviduct are considered aerobic bacteria. Furthermore, the nutrients available for microorganisms in the oviduct are very different than in the gut. Another force that may be acting to differentiate the oviduct and gut microbiomes is that the oviduct may be exposed to additional sources of bacteria, external to the cloaca, including skin and external environment.

Our results imply that not only gut pathogens can transfer from the gut to the oviduct but likely all microorganisms in the gut, including commensals. Furthermore, it is likely that presence in the oviduct per se is not a virulence characteristic of pathogens but a result of chicken physiology. Considering the fact that the oviduct microbiome is similar along its full length, ascending the oviduct is also not a virulence property specific to pathogens but again a result of chicken physiology.

It is important to note that the sperm, ova, and the fertilization process itself are all exposed at the very least to microbial DNA, if not to lipopolysaccharide (LPS) and live bacteria. It is intriguing to speculate on the possible effects of microbial derived signals on natural fertilization. Furthermore, our results imply that food additives, probiotics, and diet possibly have a much more direct effect on reproduction and egg formation than previously considered.

Moreover, the presence of gut material in the oviduct implies that gut material reaches the egg; therefore, at least bacterial products, such as LPS and DNA, may be found in the chicken egg. Thus, the chick is exposed at the very least to bacterial products. The effects of these products on the chick’s development is currently unknown.

Published work by Ding et al. showed a similarity between the microbiomes of maternal hen feces, embryos, and chick ceca, implying vertical transmission of gut bacteria (Ding et al., 2017). Here, we show the likely transfer of material from the hen’s gut to the oviduct. Once in the oviduct, bacteria are likely exposed to assault by lysozyme and other antimicrobials, which end up in the egg albumen (Gantois et al., 2009; Fang et al., 2012). Thus, it is possible that even if live bacteria reach the egg they are lysed or otherwise killed there. However, considering that *Salmonella* can survive in albumen (Lock and Board, 1992; Schoeni et al., 1995), it will be surprising if other bacteria cannot do so as well. Future research to determine the viability of oviduct bacteria is required to determine if passage to the oviduct is a dead end or the first stop to the egg and chick.

## Data Availability Statement

Sequencing files are available in SRA under BioProject PRJNA636410.

## Ethics Statement

The animal study was reviewed and approved by the Hebrew University of Jerusalem’s Ethics committee, approval no. AG-19-15897-3.

## Author Contributions

NS, NR, and EM designed the study. NS, NR, YS, ES, AN and EM performed the study. NS and EM analyzed the results and wrote the manuscript. All authors contributed to the article and approved the submitted version.

### Conflict of Interest

The authors declare that the research was conducted in the absence of any commercial or financial relationships that could be construed as a potential conflict of interest.

## Footnotes

^1^www.earthmicrobiome.org
