# Supplementary material for: Large Overlap Between the Intestinal and Reproductive Tract Microbiomes of Chickens
Source: Front Microbiol. 2020 Jul 14;11:1508. doi: 10.3389/fmicb.2020.01508 (PMC7372964; doi:10.3389/fmicb.2020.01508)
Supplement: Supplementary file 1 [file Data_Sheet_1.PDF]

<u>Figure S1</u>: Rarefaction curve of all samples. Dashed line represents the sub-sampling depth.

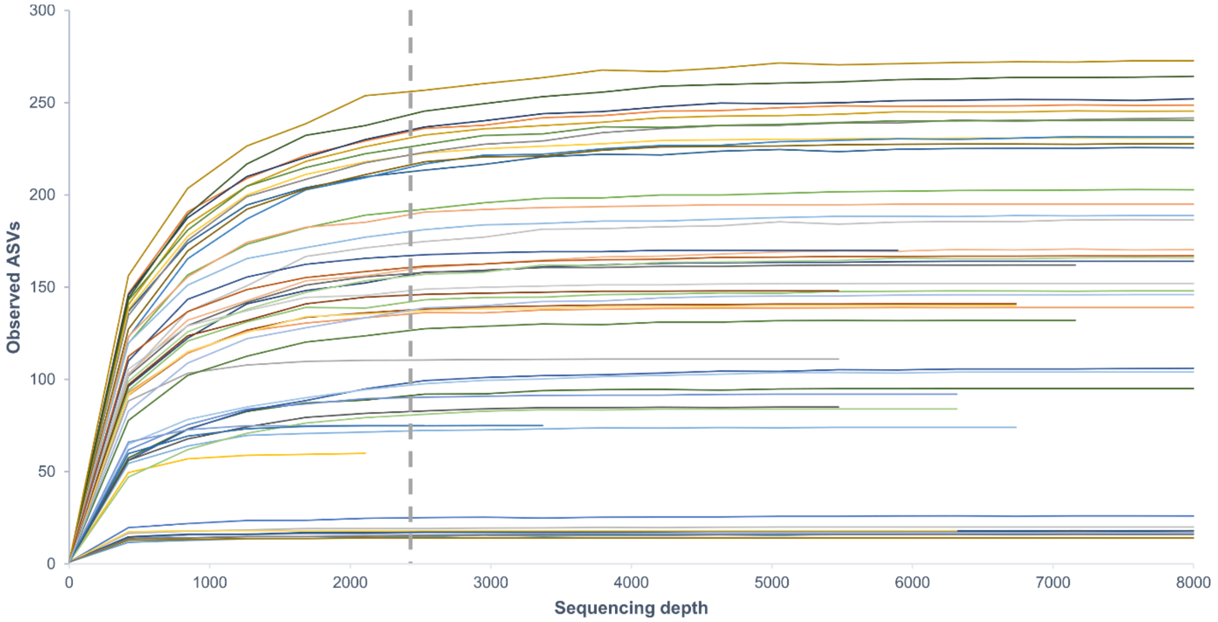

<u>Figure S2</u>: Taxonomic composition of all samples at the phylum level (A) and order level (B).

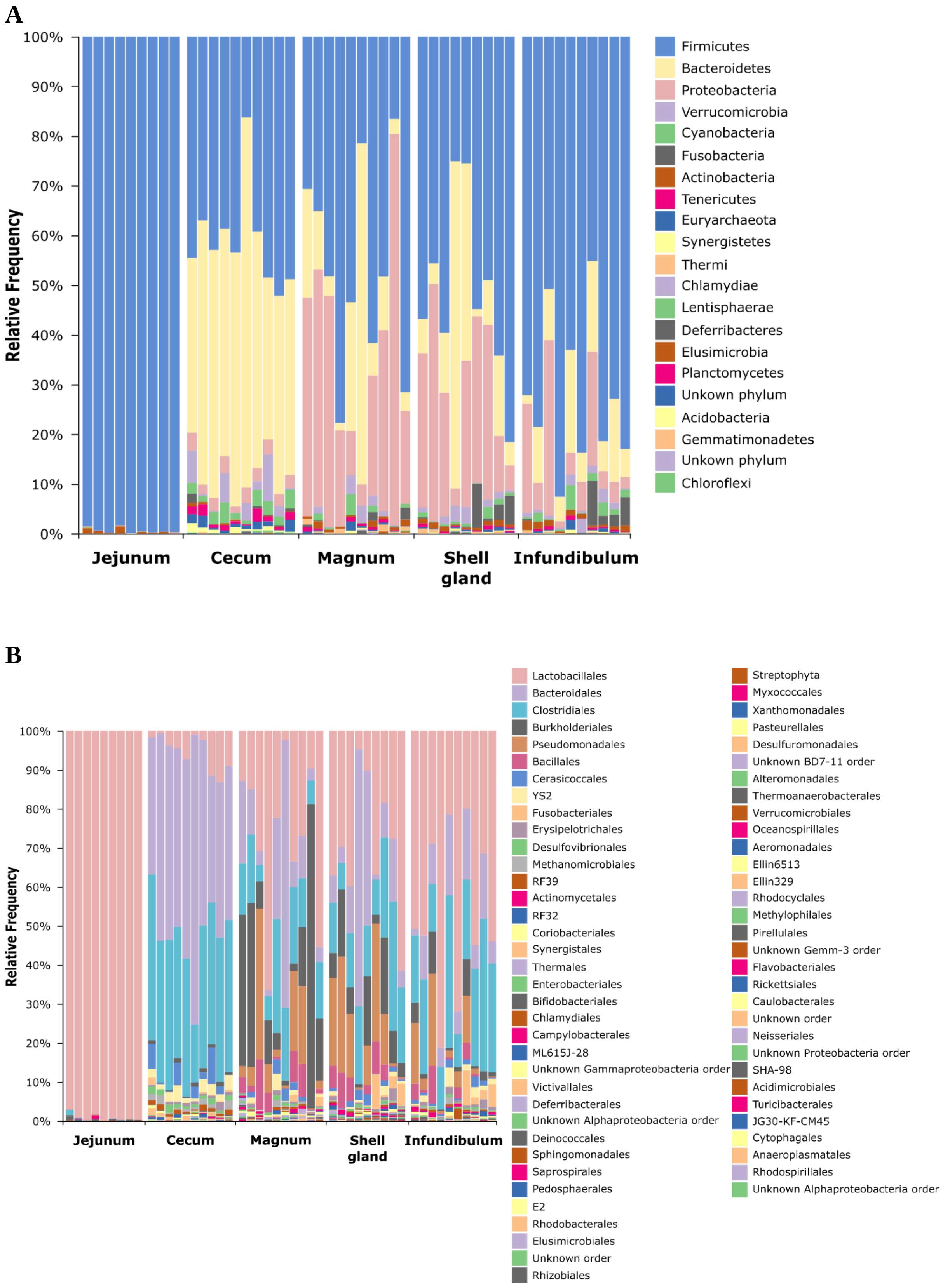

<u>Figure S3</u>: PCoA analysis of all oviduct samples based on Bray-Curtis (A) and Jaccard (B) metrics.

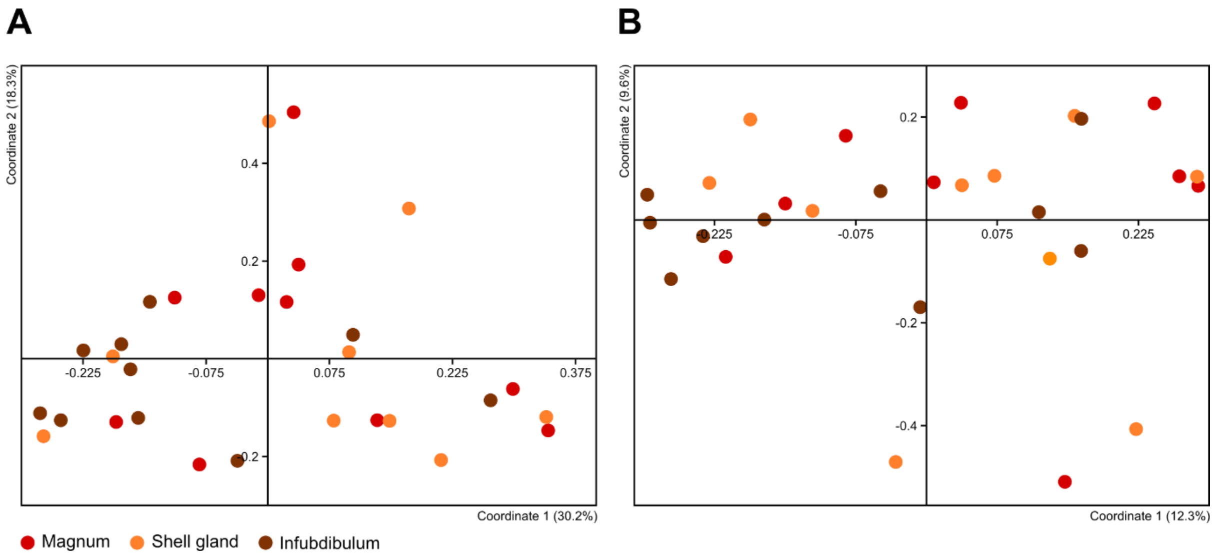

Figure S4: ASVs shared between the different sites. Individual- and pan-based analyses. The number of shared ASVs in each intersection is noted in the table, as well the percentage of the total number of ASVs in parentheses.

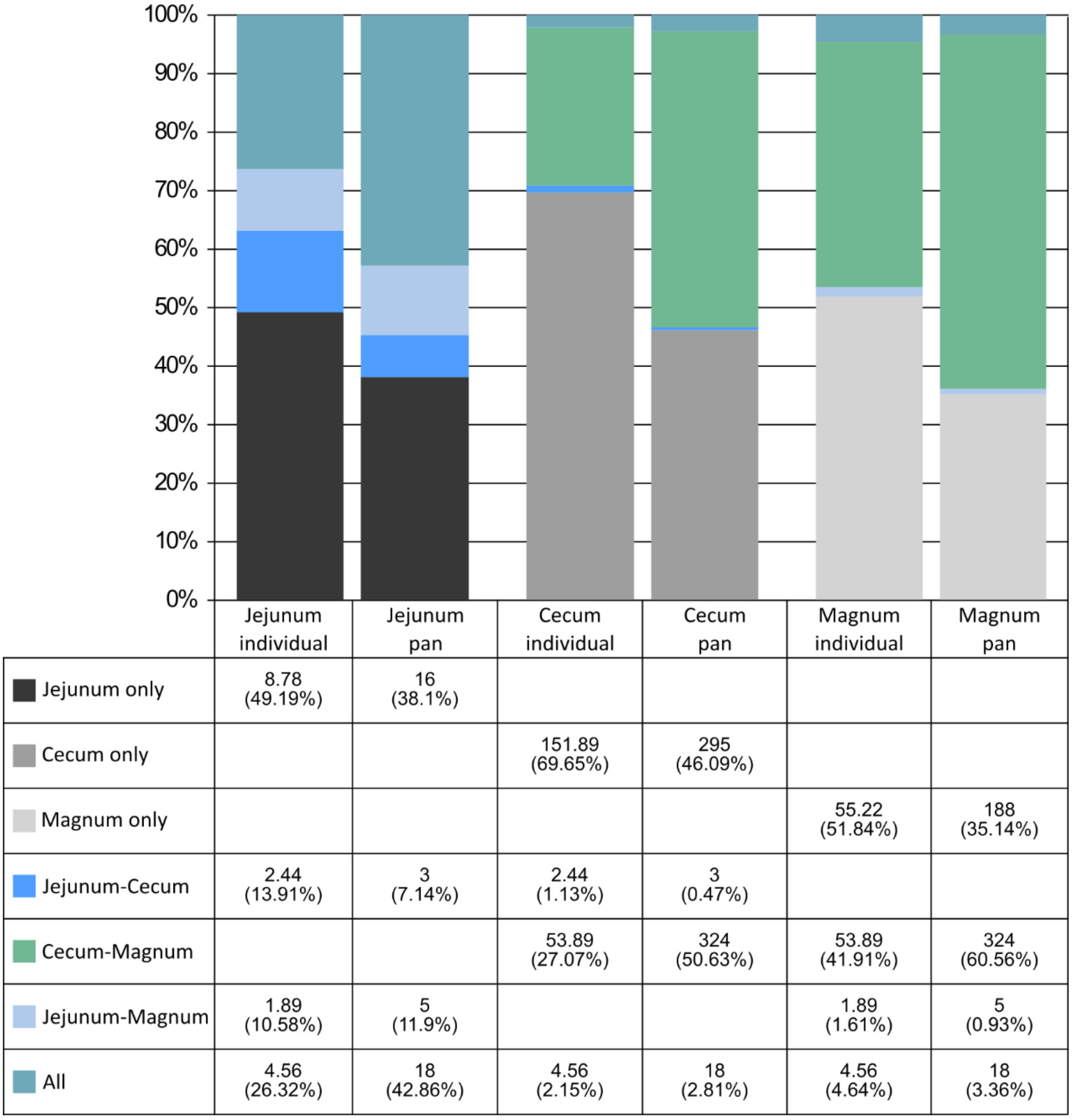

	Jejunum individual	Jejunum pan	Cecum individual	Cecum pan	Magnum individual	Magnum pan
Jejunum only	8.78 (49.19%)	16 (38.1%)				
Cecum only			151.89 (69.65%)	295 (46.09%)		
Magnum only					55.22 (51.84%)	188 (35.14%)
Jejunum-Cecum	2.44 (13.91%)	3 (7.14%)	2.44 (1.13%)	3 (0.47%)		
Cecum-Magnum			53.89 (27.07%)	324 (50.63%)	53.89 (41.91%)	324 (60.56%)
Jejunum-Magnum	1.89 (10.58%)	5 (11.9%)			1.89 (1.61%)	5 (0.93%)
All	4.56 (26.32%)	18 (42.86%)	4.56 (2.15%)	18 (2.81%)	4.56 (4.64%)	18 (3.36%)

Figure S5: The total relative abundance of ASVs in each intersection, in each of the sites, based on an individual analysis.

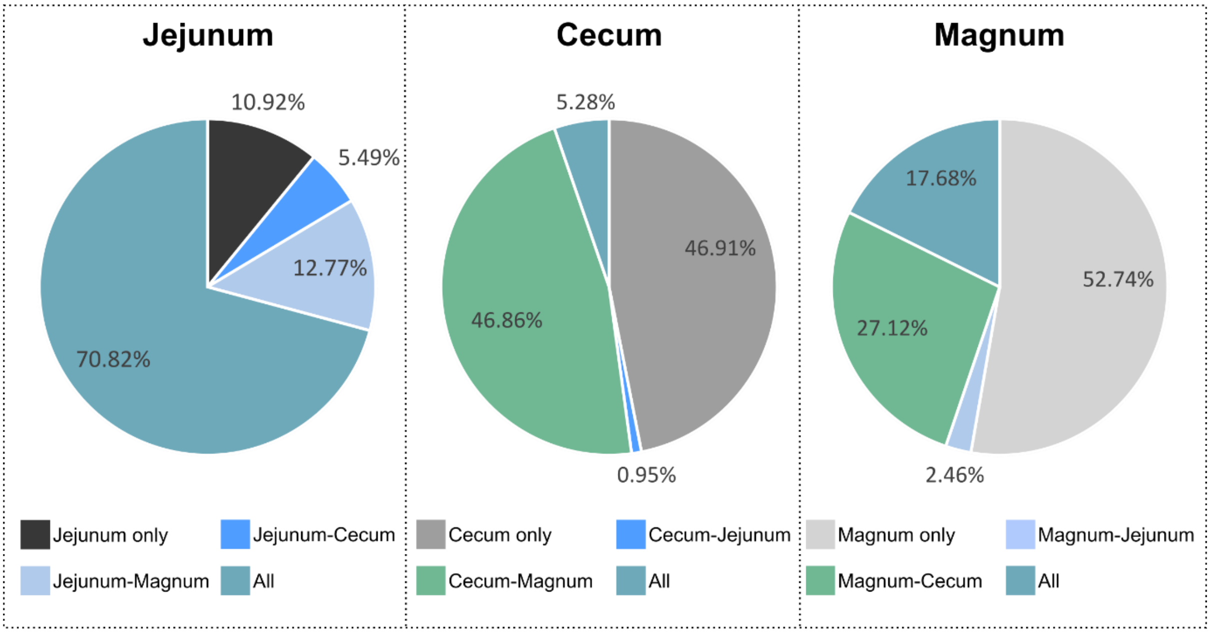

<u>Table S1</u>: ANOSIM Bonferroni-corrected *p*-values, based on Bray-Curtis metric. Significant *p*-values are highlighted.

	**Jejunum**	**Cecum**	**Magnum**	**Shell gland**	**Infundibulum**
**Jejunum**		0.001	0.002	0.002	0.001
**Cecum**	0.001		0.001	0.002	0.001
**Magnum**	0.002	0.001		1	0.575
**Shell gland**	0.002	0.002	1		0.791
**Infundibulum**	0.001	0.001	0.575	0.791	

<u>Table S2</u>: ANOSIM Bonferroni-corrected *p*-values, based on Jaccard metric. Significant *p*-values are highlighted.

	**Jejunum**	**Cecum**	**Magnum**	**Shell gland**	**Infundibulum**
**Jejunum**		0.001	0.001	0.001	0.001
**Cecum**	0.001		0.001	0.001	0.002
**Magnum**	0.001	0.001		1	0.786
**Shell gland**	0.001	0.001	1		1
**Infundibulum**	0.001	0.002	0.786	1	
